# Diagnosis of Invasive Fungal Diseases in Hematological Malignancies: A Critical Review of Evidence and Turkish Expert Opinion (TEO-2)

**DOI:** 10.4274/tjh.2014.0218

**Published:** 2014-12-05

**Authors:** Sevtap Arıkan Akdağlı, Alpay Azap, Figen Başaran Demirkazık, Beyza Ener, Sibel Aşcıoğlu Hayran, Özlem Özdemir Kumbasar, Gökhan Metan, Zekaver Odabaşı, Ömrüm Uzun, Hamdi Akan

**Affiliations:** 1 Hacettepe University Faculty of Medicine, Department of Medical Microbiology, Ankara, Turkey; 2 Ankara University Faculty of Medicine, Department of Infectious Diseases and Clinical Microbiology, Ankara, Turkey; 3 Hacettepe University Faculty of Medicine, Department of Radiology, Ankara, Turkey; 4 Uludağ University Faculty of Medicine, Department of Microbiology, Bursa, Turkey; 5 Hacettepe University Faculty of Medicine, Department of Infectious Diseases, Ankara, Turkey; 6 Ankara University Faculty of Medicine, Department of Pulmonary Diseases, Ankara, Turkey; 7 Erciyes University Faculty of Medicine, Department of Infectious Diseases and Clinical Microbiology, Kayseri, Turkey; 8 Marmara University Faculty of Medicine, Department of Infectious Diseases, İstanbul, Turkey; 9 Ankara University Faculty of Medicine, Department of Hematology, Ankara, Turkey

**Keywords:** diagnosis, invasive fungal infection, imaging, Serology

## Abstract

One of the most problematic issues in hematological malignancies is the diagnosis of invasive fungal diseases. Especially, the difficulty of mycological diagnosis and the necessity of immediate intervention in molds have led to the adoption of “surrogate markers” that do not verify but rather strongly suggest fungal infection. The markers commonly used are galactomannan (GM), beta-glucan, and imaging methods. Although there are numerous studies on these diagnostic approaches, none of these markers serve as a support for the clinician, as is the case in human immunodeficiency virus (HIV) or cytomegalovirus (CMV) infections. This paper has been prepared to explain the diagnostic tests. As molecular tests have not been standardized and are not used routinely in the clinics, they will not be mentioned here.

## Abstract (tr)

Hematolojik malignitelerin tedavisinde önemli sorunlardan birisi de invazif fungal infeksiyonların tanısıdır. Mikolojik tanıdaki zorluklar ve hızlı müdahele etme gerekliliği, küf mantarlarının tanısında dolaylı işaretleyicilerin geliştirilmesine yol açmıştır. En sık kullanılan tanı testleri galaktomannan, beta-glukan ve moleküler yöntemlerdir. Her ne kadar bu tanı yöntemleri ile ilgili çok sayıda araştırma yapılmakta ise de, hiçbiri HIV ya da CMV’de olduğu gibi doğrudan yarar sağlamamaktadır. Tanı ile ilgili bu yazı klinisyene eldeki araçları kullanma konusunda yol göstermeyi amaçlamaktadır. Moleküler testlere henüz standardize edilmediği ve klinik kullanıma girmediği için bu yazıda değinilmeyecektir.

## INTRODUCTION

One of the most problematic issues in hematological malignancies is the diagnosis of invasive fungal diseases. In particular, the difficulty of mycological diagnosis and the necessity of immediate intervention in cases of molds have led to the adoption of “surrogate markers” that do not verify but rather strongly suggest fungal infection. The markers commonly used are galactomannan (GM), beta-glucan, and imaging methods. Although there are numerous studies on these diagnostic approaches, none of these markers serve as support for the clinician, as in human immunodeficiency virus (HIV) or cytomegalovirus (CMV) infections. This paper has been prepared to explain the diagnostic tests. As molecular tests have not been standardized and are not used routinely in clinics, they will not be mentioned here.

**Conventional Microbiological Diagnostic Methods**

Clinical manifestations of invasive fungal infections (IFIs) generally are not agent-specific. Therefore, radiological, histopathological, and microbiological features are important in diagnosis. Although the radiological and histopathological findings are very important and provide possible evidence, the causative agent should be grown in culture and preferably be identified to the species level for definitive diagnosis. Accordingly, conventional (classical) microbiological diagnostic methods remain the gold standard in the diagnosis of IFIs [1,2,3,4].

**Conventional Microbiological Diagnostic Methods and Current Guidelines**

Direct microscopic examination and culture of the clinical samples constitute the conventional microbiological diagnostic methods.

**Direct Microscopic Examination**

Direct microscopic examination of the sample is a rapid and practical method that provides a chance for a possible pre-diagnosis. The results of direct microscopy also allow the interpretation of culture contamination in specimens from which a mold is isolated later. The major factors affecting the sensitivity of direct microscopic examination include the collection of a sufficient volume of a suitable clinical sample, application of staining methods within the frame of related principles, and performance of microscopic examination by experienced people on a sample involving the whole specimen. The sensitivity of direct microscopy widely varies depending on these factors. In order to evaluate the microscopic structures of fungi, different stains and wet coating methods can be used. Furthermore, very important evidence for the diagnosis of fungal infections can be acquired by histopathological evaluation of the biopsy samples. The staining methods used in microbiological and histopathological evaluation for the diagnosis of opportunistic mycoses and the goals of use are summarized in Table 1 [5,6].

**Culture**

The isolation of fungi in culture is a conventional method, which remains the gold standard in providing the definitive diagnosis of IFIs. The collection of sufficient amounts of appropriate sample, rapid transport of the specimen to the laboratory, the informing of the laboratory of the tentative clinical diagnosis, and performance of the laboratory examinations without delay according to standard recommendations are among the main factors affecting the success of a culture [5,6,7]. The culture method carries some disadvantages along with important advantages; therefore, the use of additional serological diagnostic methods, particularly those aimed at early diagnosis, is recommended along with culture. The advantages and disadvantages related to the culture method [4,8,9] are presented in Table 2.

**Available Guidelines Related to the Microbiological Diagnosis of Invasive Fungal Infections**

In previous years, guidelines for or involving sections on fungal infection diagnosis have been published. The names and major content of these guidelines are summarized in Table 3.

Recommendations for Direct Microscopic Examination and Culture Applications

Direct microscopic examination and culture are the main indispensable methods for IFI diagnosis. Direct microscopic examination, due to its advantages such as being rapid and providing a possible pre-diagnosis, should necessarily be performed. The culture method, with advantages such as identification of the causative agent at species level and accordingly prediction of antifungal susceptibility profile at the species level further allowing performance of antifungal susceptibility tests for that strain, is the gold standard that should be performed as the fundamental diagnostic method whenever possible. The use of culture in diagnosis has also received the highest recommendation level as the main diagnostic method in the current guidelines.

**Diagnosis of Fungemia: Isolation of Fungi in Blood Cultures**

Principles of collecting samples for the isolation of fungi in blood culture are the same as the standard principles of blood culture [3]. Blood culture is an important method for the isolation of Candida, Fusarium, Scedosporium, thermally dimorphic fungi causing endemic mycosis, and the other yeasts with clinical importance [Blastoschizomyces (Saprochaete), Rhodotorula, Trichosporon, etc.] and for the diagnosis of infections caused by these fungi. If the fact that Turkey is not in an endemic region for thermally dimorphic fungi is considered, excluding possible travel-related infections, isolation of fungi causing endemic mycosis from blood does not carry primary importance. Fungi other than those causing endemic mycoses and expected to be isolated in blood culture can be isolated in blood culture at varying rates in our country, with Candida spp. being the leading isolate. However, the sensitivity of blood culture in candidemia diagnosis is generally below the desired level. Autopsy studies show that candidemia can be diagnosed by blood culture in 50-70% of cases [10,11].

Different methods or parameters are being tested to enhance the sensitivity of blood culture in fungemia diagnosis, and the leading methods are lysis centrifugation method and use of fungal media. The lysis centrifugation method is especially recommended for the identification of dimorphic fungi in blood culture [12]. However, studies published in previous years, particularly those involving open systems, reported data indicating that the use of the lysis centrifugation method might increase the risk of contamination [4,13]. Beyond that, as the lysis centrifugation method has been reported to be mainly useful in the diagnosis of dimorphic fungi, it does not carry primary importance in Turkey. On the other hand, use of fungal media has been observed to be important not only for the isolation of fungi causing endemic mycosis, but also for the growth duration of yeasts (especially for Candida glabrata); use of fungal media has been reported to shorten the isolation period [14,15]. This finding is important, as C. glabrata is isolated in varying rates in blood culture at different centers in our country.

When aerobic culture bottles of different automated systems are used for the isolation of fungi, the isolation rate of C. glabrata has been demonstrated to differ according to the automated system used, and the use of fungal media is particularly recommended with systems yielding low rates of isolation [16]. Other than that, study data are available showing that the use of fungal media can be beneficial in intensive care or hematology cases, where yeast growth in blood may be found together with resistant or multiple bacterial growth [14]. In light of these data, although current guidelines primarily stipulate the use of automated, validated blood culture systems, they emphasize that the sensitivity of the system may change depending on the species studied and the system itself [3]. The knowledge that the use of blood culture bottles containing fungal media may shorten the growth period and the recommendation that the decision related to the use of these media should be made by the related center also appear in the guidelines [12]. Additionally, the Infectious Diseases Society of America (IDSA) and the American Society for Microbiology (ASM) guidelines offer the use of 2 aerobic blood culture bottles instead of a single bottle for inoculation as an alternative to inoculation into fungal media, if yeast fungemia is suspected [17].

**Recommendation for the Isolation of Fungi from Blood Culture**

If the conditions of our country are taken into account, fungi likely to be isolated from blood include Candida spp., the other yeasts, and Fusarium spp. In accordance with the recommendations of the guidelines and the studies performed using Candida spp., it is recommended that the decision of using fungal media for inoculation should be made considering the properties of the automated system and the patient population of that center. 

**Routine Antifungal Susceptibility Testing and Recommendations**

Antifungal susceptibility testing should be performed with certain indications to direct antifungal treatment, not for all fungal strains isolated in routine practice. The indications of antifungal susceptibility testing have gained accuracy mostly for Candida strains, and comprehensive studies on other fungi, especially Aspergillus, are also being carried out. The indications for antifungal susceptibility testing include strains isolated from normally sterile sites, unresponsiveness to antifungal treatment, history of previous antifungal treatment use, and decreased susceptibility to antifungal drug(s) or isolates belonging to a resistant strain. Antifungal susceptibility testing should be performed and interpreted using reference methods for antifungal susceptibility tests from the Clinical and Laboratory Standards Institute (CLSI) or the European Committee on Antibiotic Susceptibility Testing (EUCAST) by staff and centers experienced in antifungal susceptibility testing and educated in mycology [3,4,18,19,20,21].

**Serological Tests**

Serological tests, developed to aid in early diagnosis and recommended to be used along with conventional methods, are another test group that carries importance in the microbiological diagnosis of IFIs. Currently, the important serological tests used in routine practice are the GM antigen test, beta-glucan test, cryptococcal antigen test, and combined mannan-anti-mannan testing.

**Galactomannan Test**

GM is a molecule composed of mannan and galactofuranose polymers found in the cell wall of Aspergillus spp. and some other molds (Penicillium and Fusarium spp.) [28,29]. It may be released into the environment during active growth of the fungus. It has been shown in several studies that determination of GM in serum, bronchoalveolar lavage (BAL) fluid, and other samples by a commercial kit using the sandwich enzyme immunoassay (EIA) method (Bio-Rad Laboratories, Marne-La-Coquette, France) is beneficial in the diagnosis of invasive aspergillosis (IA) [30,31,32,33]. The results are read by a spectrophotometer and expressed as optical density index. It is strongly (AII) recommended by the ECIL that it be performed as a screening test in bone marrow transplants and in patients with hematological malignancies in whom the incidence of IA is high (5-15%). Again, strong evidence (AII level) supports screening to be performed at 2- to 3-day intervals at least twice a week, and an optical density index of >0.7 in a single sample and an optical density index of >0.5 in 2 consecutive samples suggests IA [22]. It is important to continue screening in patients diagnosed with IA by GM testing (BII) and it was demonstrated in studies that not observing a decrease in optical density may be related to poor prognosis [34]. Additionally, revised European Organization for Research and Treatment of Cancer/Mycoses Study Group (EORTC/MSG) criteria emphasize that probable IA diagnosis can be made by GM positivity even if conventional microbiological evidence is not available [35].

There are several factors affecting the performance of the GM test. These factors are summarized in Table 4. It is emphasized that there may be false negative results in non-neutropenic patients receiving antifungal prophylaxis/treatment and in local infections, and false positive results in patients with Fusarium infections and in those receiving semi-synthetic β-lactam antibiotics [28,36,37,38,39,40]. However, recent studies showed a very low rate of false positive GM related to piperacillin/tazobactam, which was considered as the most common antibiotic associated with false GM results [41,42,43]. Such kind of false positive results can be avoided by obtaining the serum sample just before the next dose of semi-synthetic β-lactam antibiotics [44].

Other than serum, GM testing can be performed in BAL fluid. It was reported in all meta-analyses that the sensitivity of BAL GM is higher and the specificity is lower than that of serum GM testing [45,46,47]. It is recommended to perform GM analysis in bronchoscopic samples of patients at high risk of IA as its negative predictive value is high. It was pointed out that a BAL GM optical index of <0.5 excludes IA diagnosis, whereas an optical index of >3 supports definitive diagnosis [48,49]. The values between these 2 limits are debatable. However, it is recommended in meta-analyses that the cutoff index value be taken as 1.5 in patients with hematological malignancies and 1 in mixed patient populations [45,46]. A study comparing culture and GM in BAL fluid samples emphasized that growth might be expected when the optical index is >1, and a correlation with clinical studies was also demonstrated [50].

There are also various factors affecting the performance of BAL GM testing. While the use of antifungal drugs active in molds leads to an apparent decrease in sensitivity, no significant difference could be found related to Aspergillus spp. Furthermore, there is no difference in the sensitivity of BAL GM in patients with or without neutropenia [45,50]. Similar to serum GM testing, the use of semi-synthetic β-lactam antibiotics may lead to false positive results [45]. The standardization of bronchoscopy and bronchoscopic material is the most urgent problem that should be solved regarding BAL GM testing.

Conclusively, GM is a biomarker that can be used along with clinical, radiological, and conventional microbiological tests in IA diagnosis. In centers where high-risk patients (bone marrow transplants and patients with hematological malignancies) are followed, if a result can be achieved in 24 h and if computed tomography (CT) is available, it is recommended to be used as a screening test. In Turkey, GM testing twice a week for hospitalized patients is covered by reimbursement. Multidisciplinary follow-up is needed in high-risk patients and performance of bronchoscopy and BAL GM analysis after CT is important to exclude or support the diagnosis. There is a decrease in the sensitivity of GM testing, and especially serum GM, in patients receiving prophylaxis. It can be used as a diagnostic test when clinical findings develop in this kind of patient or at centers where scanning cannot be performed. However, it should not be forgotten that this is a supplementary test that should always be used together with other data.

**1,3-beta-D-Glucan Test**

The 1,3-beta-D-glucan (BG) test is one of the promising non-culture-based early diagnostic tests for the diagnosis of IFIs. BG is a cell wall component of many fungi, especially Candida and Aspergillus spp. [51,52]. This test is based on the reaction of BG with factor G of the horseshoe crab coagulation cascade. In a preliminary study of 30 candidemic patients and 30 healthy controls, a serum BG level of 60 pg/mL was chosen as the cutoff. After determining this cutoff value, the BG test was evaluated in 283 patients with acute myeloid leukemia or myelodysplastic syndrome and the sensitivity, specificity, and negative predictive value of the test in proven and probable IFIs was 100%, 90%, and 100%, respectively [51]. Later, a cutoff value of 80 pg/mL, which is the currently accepted positive cutoff value for the test, was found to have better specificity in a multicenter study [53]. In an in vitro study, reactivity of 127 clinical fungal isolates belonging to 40 different genera was evaluated with the Glucatell assay [54]. Compared with the reactivity of Aspergillus spp. with the BG test, Bipolaris spicifera, Sporothrix schenckii, Wangiella dermatitidis, and Penicillium marneffei isolates showed stronger reactivity and Paecilomyces spp., Scopulariopsis spp., Fusarium spp., Phialophora verrucosa, and Exophiala jeanselmei showed some reactivity. Among the tested clinical yeast isolates, Saccharomyces spp., Rhodotorula rubra, and Trichosporon asahii were found to have similar test reactivity when compared to Candida spp. In a clinical study, sensitivity of the BG test for the diagnosis of Candida parapsilosis was found to be lower compared to other Candida spp. (78% vs. 90%, respectively) [53]. This test can also be positive in Pneumocystis jirovecii infections and the sensitivity of the test is very high: 96%, with a specificity of 84% [55]. The performance of the test in BAL fluid was also evaluated; however, the sensitivity of detecting an IFI using BAL specimens was not significantly increased over testing of serum alone [56].

The BG test is usually not positive in the case of fungal colonization, and detection of circulating BG levels may be used as a surrogate marker not only for diagnosing IFIs but also for assessing the effectiveness of therapy [51,57]. Diagnostic levels and serum kinetics of the BG test were found to be similar when compared to the GM antigen test for the diagnosis of IA in neutropenic patients [57]. In the same study, the combination of BG and GM antigen tests improved the specificity and positive predictive value to 100%. In another clinical study performed in hematologic malignancy patients with IA, the sensitivity of the BG test and GM antigen test was 67% and 38%, respectively [39]. Interestingly, diagnostic accuracy of the GM antigen test was 13% per serum sample in A. fumigatus-positive patients compared to 49% positivity in patients with non-fumigatus Aspergillus spp.; BG test positivity was similar between A. fumigatus and non-fumigatus Aspergillus spp.

The overall pooled sensitivity and specificity of the test for the diagnosis of IFIs is 77% and 85% [58]. Those values seem promising, but there are no prospective studies evaluating the use of the BG test in the diagnosis and treatment of IFIs in hematology-oncology patients. Two consecutive positive antigenemia assays have very high specificity, positive predictive value, and negative predictive value but the sensitivity is not satisfactory, so the BG test needs to be combined with clinical, radiological, and microbiological findings [59]. Ideal cutoff values and timing of serum samples (like 2 or 3 times a week) should be determined. Currently, use of the test at least twice a week is moderately recommended in some guidelines for the screening or diagnosis of invasive Aspergillus and Candida infections [3,22]. Table 5 summarizes the differences between the GM and BG tests.

**Cryptococcal Antigen Tests**

The polysaccharide capsule that surrounds Cryptococcus neoformans is the target structure in serological tests, due to its antigenic structure. LA and EIA are the methods accepted in the serological diagnosis of cryptococcosis [22]. The concordance of LA and EIA kits developed by different companies was found to be higher than 90% [62]. In a systematic review performed after January 1998 evaluating 7 studies, 6 of which were retrospective, it was reported that cerebrospinal fluid (CSF) antigen test sensitivity in cryptococcal meningitis was 97% [63]. Although the effect of CSF antigen titer on disease prognosis has not been clearly defined, a CSF or serum antigen titer higher than 1/512 was associated with disseminated infection and unresponsiveness to treatment [64,65]. Antigen titer, tested again at least 7 days after the first test, was reported to decrease by 4-fold in patients with treatment response [66]. The sensitivity of the serum antigen test was reported to be between 62% and 67% in pulmonary cryptococcosis and cases of other organ involvement. Antigen test specificity in both serum and CSF samples was determined to be 93-100% [64,65,66,67]. Serum antigen positivity has been reported in HIV-positive patients with disseminated disease. It should be taken into account that serum antigen tests may give false negative results in cancer patients [22]. False positive test results at low titers were reported more frequently in LA tests than in EIA. False negative results are more likely with kits where clinical samples are not pre-treated with pronase [68]. In a study performed between January 1989 and December 1999, 3828 CSF cryptococcal antigen tests of cancer patients were evaluated, and positive results were reported in 12 patients (0.3%). Six of these patients were found to have other conditions involving the central nervous system and it was recommended that test results of such patients be interpreted carefully because of probable false positive results [69]. Cryptococcal antigen testing in serum and CSF samples of patients with cryptococcal meningitis and disseminated cryptococcosis is recommended with an AII evidence level, and the use of the test in following treatment response is recommended with a CIII evidence level by the ECIL [22].

Although cryptococcosis is not a common disease in Turkey, cryptococcal meningitis should be included in the differential diagnosis of central nervous system disorders of stem cell transplants or patients treated with intense chemotherapy regimens, and cryptococcal antigen testing should be performed on the CSF samples of such patients.

**Mannan/Anti-Mannan Tests**

Determination of mannan antigen (Mn) and anti-mannan antibody (A-Mn) by ELISA method has become one of the most frequently used serological methods in the diagnosis of invasive candidiasis [22]. A systematic review, evaluating 14 studies (13 retrospective, 1 prospective) including a total of 1220 patients (453 invasive candidiasis cases, 767 controls), reported that when performed separately the sensitivity of Mn and A-Mn tests was 58% and 59%, respectively, whereas the specificity was 93% for Mn and 83% for A-Mn. However, if the positivity of one of these tests (Mn/A-Mn combination) is considered to be sufficient for diagnosis, sensitivity reaches 83% without a significant decrease in specificity (86%) [70]. In 73% of 45 patients with candidemia, at least one of the tests yielded positive results 6-7 days before the culture results were available, and in 21 patients who developed hepatosplenic candidiasis, a positive result was achieved approximately 16 days before the culture results [71,72]. Among Candida spp., the highest sensitivity was achieved for Candida albicans [70]. Seven of the studies evaluated in this review were performed in cancer patients, while the other 7 studies were carried out in intensive care units and surgery clinics. The heterogeneity of the methods used in the studies is intriguing. When the hematology-oncology patients were evaluated as a subgroup, the sensitivity of the test was 71-100% and the specificity ranged between 53% and 92% [70]. It was reported that response to Mn was especially significant after resolution of neutropenia [73]. A study that compared the Mn, A-Mn, and BG test results in 56 candidemia patients, including 9 stem cell transplants or patients with hematological malignancies and 12 patients with solid tumors, reported the sensitivity of the tests as 58.9%, 62.5%, and 87.5%, respectively, and the specificity as 97.5%, 65%, and 85.5%, respectively. While the diagnostic sensitivity of the Mn/A-Mn combination increased to 89.3%, specificity remained at 63% [74]. ECIL guidelines recommend the use of Mn and A-Mn together with an evidence level of BII, and the use of Mn/A-Mn combination in the diagnosis of hepatosplenic candidiasis with an evidence level of BIII and in the diagnosis of candidemia in hematology-oncology patients with evidence level CII. In the same guidelines, the use of the BG test in the diagnosis of invasive fungal disease is recommended at the BII evidence level [22]. In the ESCMID guidelines for the diagnosis of Candida infections, the use of Mn/A-Mn combination is recommended with the same evidence level as the use of BG.

Recommendation

Considering the economic burden of performing 2 separate tests for the Mn/A-Mn combination, the similar clinical pictures in cancer patients with several other fungi besides Candida, and the panfungal character of the BG test contributing to the diagnosis of most of these conditions, the use of the BG test seems to be more suitable in neutropenic cancer patients in Turkey. Each center should decide which test they will use according to the incidence of invasive fungal disease, technical infrastructure, and target patient groups.

**Radiological Diagnosis of Invasive Fungal Infections**

CT is a more sensitive imaging method than chest X-ray in the diagnosis and differential diagnosis of pulmonary infections in immunosuppressed patients. CT is particularly indicated if chest X-ray is normal or near normal in patients suspected of pulmonary infections [75,76]. Currently, the entire lung can be scanned in a single breath-hold with multislice spiral CT (MSCT) systems. Continuously acquired slices of 5 mm in thickness can be used in routine evaluation. However, in order to identify the halo sign seen in fungal infections, thinner slices (1-2 mm) should be used in evaluation. The resolution of thin slices obtained with MSCT is sufficient to determine parenchymal lesions, and the use of classical high-resolution CT (HRCT) technique before MSCT is not necessary. MSCT especially provides better imaging than HRCT in patients who cannot hold their breath. However, while HRCT scans obtained in the prone position are helpful in the diagnosis of patients suspected of early fibrosis, HRCT scans obtained in expiration phase aid in the diagnosis of patients suspected of bronchiolitis obliterans [75].

There is no need to use intravenous contrast media for MSCT for detection and differential diagnosis of parenchymal infections in immunosuppressed patients. However, at the later stages, if there is suspicion of a rare vascular complication such as mycotic pulmonary artery aneurysm or aortic aneurysm, intravenous contrast media should be used. Intravenous contrast media are used for liver and spleen imaging of patients suspected of disseminated candidiasis [75].

Symptoms of IFIs are not specific in immunosuppressed patients and differential diagnosis from infections caused by other microorganisms and noninfectious causes should be made. Therefore, radiologists should be informed in detail about the history and clinical-laboratory findings (primary disease, history of chemotherapy or radiotherapy, fever, respiratory distress, neutropenia, etc.) of the patients when they are evaluating thoracic CT scans.

**Thorax CT Findings in Invasive Fungal Infections**

Chest X-ray findings in immunosuppressed neutropenic patients are nonspecific and the assessment should be done by CT. A nodule is detected in the thorax CT scans of 82%-94% of patients with IFIs; the nodule is generally 1 cm or more in diameter. Nodules are more frequent in IFIs than in bacterial or viral infections. In particular, the “halo” sign at the periphery of the nodule or the “air crescent” sign within the nodule are findings in favor of IFIs [75,76,77].

**Figure 1:**
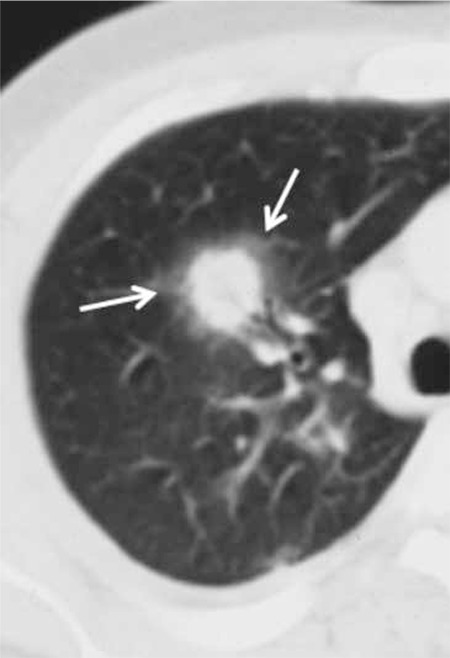
Invasive Aspergillus infection showing a nodule with a halo sign at the periphery (arrows).

The halo sign is a ground-glass opacity surrounding a nodule or a mass in CT [78]. It was first described as a sign of hemorrhage around the foci of IA (Figure 1). Besides Aspergillus infections, it may be seen in Candida infections and mucormycosis. The halo sign is nonspecific, and it may be seen in other nodules with hemorrhage or as an in situ adenocarcinoma at the periphery of invasive adenocarcinoma. The halo sign is seen more frequently in patients with hematological malignancies and stem cell transplants than in solid organ transplants. While a halo sign is found in the majority of patients with invasive Aspergillus infection in the first days of infection (88-96%), its prevalence decreases with the progression of disease and it is present in 18-19% of patients at the end of 2 weeks [79,80].

The reversed halo sign, seen in approximately 4% of IFIs, is defined as a focal, rounded area of ground-glass opacity surrounded by a complete or nearly complete ring of consolidation. While it is seen in 19% of mucormycosis cases, its prevalence is lower than 1% in Aspergillus infections. Therefore, the reversed halo sign shows that mucormycosis should be considered, and it aids in selecting the appropriate antifungal agent in treatment [81].

**Figure 2:**
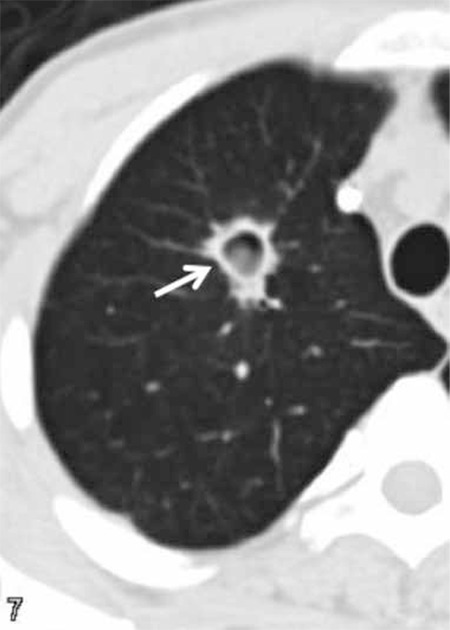
Aspergillus infection showing a nodule with cavitation and air crescent sign (arrow).

**Figure 3:**
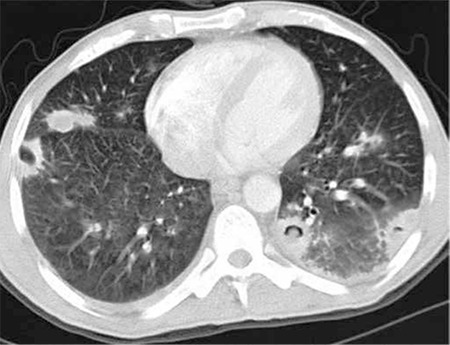
Invasive aspergillosis infection showing nodular densities, some with cavitation and air crescent signs.

At 2-3 weeks after commencement of treatment, along with the resolution of neutropenia, cavitation develops within the consolidation or within the nodule. Cavitation usually shows that prognosis is good. An air crescent sign may be present or absent. The air crescent sign is a crescent-like air space within the cavity, separating a mass from the cavity wall [78] (Figures 2 and 3).

Characteristically, it shows that the infarcted lung is separated from the wall in IA. However, it can be seen in conditions such as tuberculosis infection, Wegener granulomatosis, lung cancer, and hemorrhage within the cavity. Although the air crescent sign is a rare finding in the early stages of invasive Aspergillus infections, the prevalence increases with the progression of disease [75].

The nodule may grow within 10 days of commencing antifungal treatment in IFIs. Some researchers explained this by the gathering of immune cells along with the improvement in bone marrow. Hence, even though the nodule enlarges in the first 10 days of treatment, if the GM level is low and the number of neutrophils is high, it is recommended not to change the antifungal treatment [79,80].

Aspergillus bronchopneumonia develops in approximately 10% of invasive Aspergillus infections. This infection, also known as airway IA, is characterized by the presence of Aspergillus organisms deep in the airway basement membrane. Clinical manifestations include bronchopneumonia, tracheobronchitis, and bronchiolitis. There may be consolidation areas predominantly in the peribronchiolar regions. Lobar consolidations may be rarely seen. Generally, there are no radiological findings in acute tracheobronchitis. In rare cases, there may be thickening of tracheal or bronchial walls. “Tree-in-bud” signs, which indicate endobronchiolar or peribronchiolar disease, and centrilobular nodules may be seen in bronchiolitis. Centrilobular nodules may also be detected in endobronchial spread of tuberculosis, atypical tuberculosis infections, viral pneumonias, and mycoplasma pneumonias. Therefore, when bronchiolitis findings are detected in a CT scan, primarily infections related to bacterial or viral microorganisms are considered, as they are more frequent [76].

**Figure 4:**
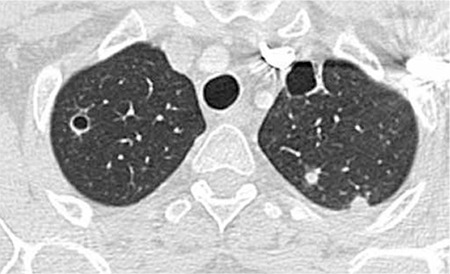
Mucormycosis showing nodular densities, some with cavitation.

There is pulmonary involvement in 30% of cases of mucormycosis. Radiological findings are not specific; consolidation, nodule, mass, cavitation, or abscess development may be seen (Figure 4) [76].

**Figure 5:**
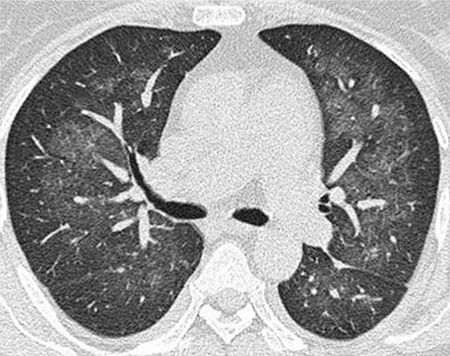
Pneumocystis pneumonia showing bilateral, perihilar ground-glass opacities; the lung periphery is spared.

In Pneumocystis jiroveci pneumonia, although chest X-rays are normal, perihilar ground-glass opacities showing patchy or geographic distribution can be identified in HRCT (Figure 5). Frequently, there is interlobular septal thickening. Cystic changes can be seen in the lungs of acquired immunodeficiency syndrome (AIDS) patients receiving prophylaxis [76].

**The Diagnosis of Extra-Pulmonary Invasive Fungal Infections**

CT is the method of choice in suspicion of IFIs in the paranasal sinuses, and magnetic resonance imaging (MRI) should be performed in cases of suspicion of central nervous system involvement. Ultrasound, CT, or MRI can be used for examining the abdomen in disseminated candidiasis; typically small abscesses with a target-like appearance are seen in the spleen and liver [82].

**Interpretation**

Although currently there are various opportunities in the diagnosis of invasive fungal diseases, their implementation is somewhat difficult. None of these tests can be used to directly diagnose invasive fungal disease and they generally carry more meaning when they are used together. Radiological diagnosis seems to remain the most rapid and easy method to use; however, it carries a risk of false positive results. Serological tests such as GM, BG, and Mn/A-Mn are not available at all institutions and carry a risk of false positive or negative results, and delays in reporting make it difficult to use them in diagnosis. Molecular approaches are not yet recommended, as they are not standardized, carry a risk of false positive results, and do not have widespread use; however, in the very near future they have the potential to hold an important place in diagnosis.

**Conflict of Interest Statement**

The authors of this paper have no conflicts of interest, including specific financial interests, relationships, and/or affiliations relevant to the subject matter or materials included.

## Figures and Tables

**Table 1:**
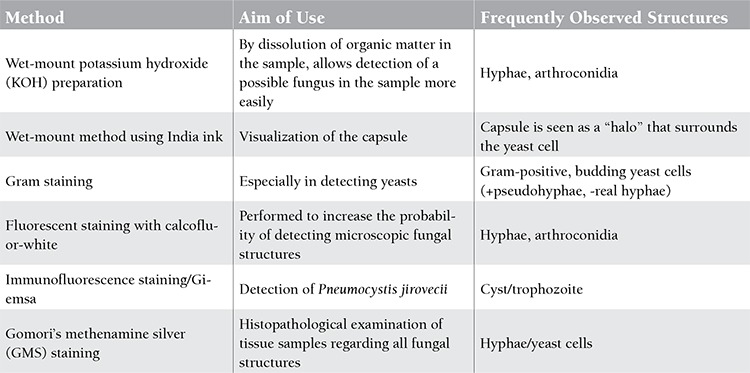
Direct microscopic examination methods used in the diagnosis of opportunistic mycoses [5,6].

**Table 2:**
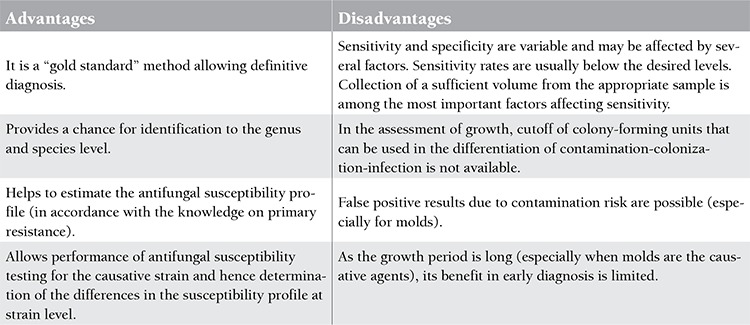
The advantages and disadvantages of culture method in the diagnosis of invasive fungal infections [4,8,9].

**Table 3:**
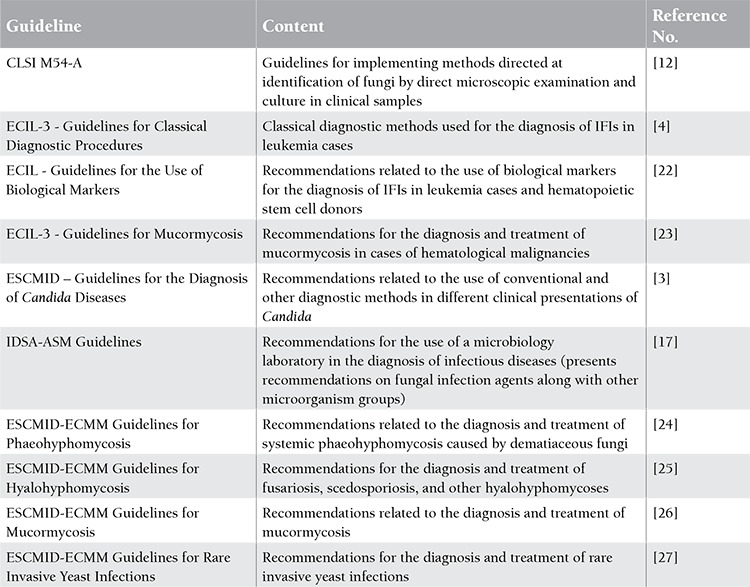
The advantages and disadvantages of culture method in the diagnosis of invasive fungal infections [4,8,9].

**Table 4:**
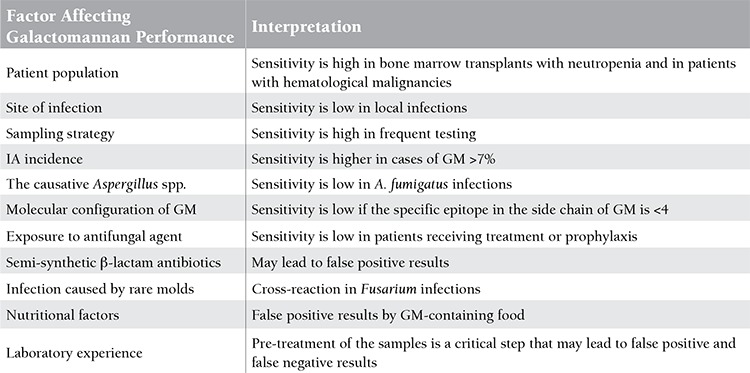
Factors affecting the performance of galactomannan testing.

**Table 5:**
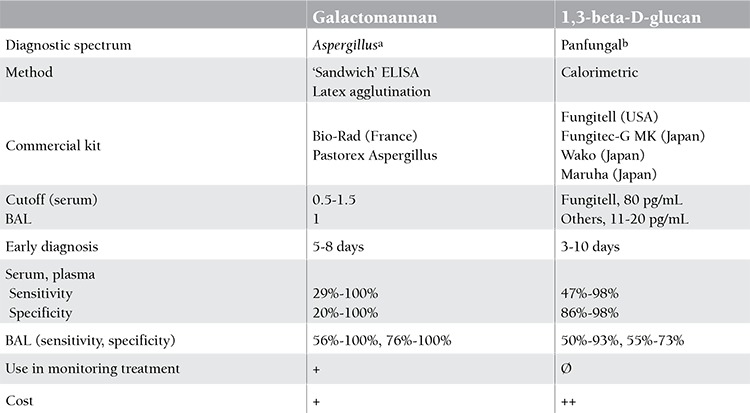
Basic characteristics of galactomannan and 1,3-beta-D-glucan tests including bronchoalveolar lavage [56,60,61].

## References

[1] Perfect JR (2003). Fungal diagnosis: how do we do it and can we do better?. Curr Med Res Opin.

[2] Rueping MJ, Vehreschild JJ, Cornely OA (2009). Invasive candidiasis and candidemia: from current opinions to future perspectives. Expert Opin Investig Drugs.

[3] Cuenca-Estrella M, Verweij PE, Arendrup MC, Arikan-Akdagli S, Bille J, Donnelly JP, Jensen HE, Lass-Flörl C, Richardson MD, Akova M, Bassetti M, Calandra T, Castagnola E, Cornely OA, Garbino J, Groll AH, Herbrecht R, Hope WW, Kullberg BJ, Lortholary O, Meersseman W, Petrikkos G, Roilides E, Viscoli C (2012). ESCMID guideline for the diagnosis and management of Candida diseases 2012: diagnostic procedures. Clin Microbiol Infect.

[4] Arendrup MC, Bille J, Dannaoui E, Ruhnke M, Heussel CP, Kibbler C (2012). ECIL-3 classical diagnostic procedures for the diagnosis of invasive fungal diseases in patients with leukaemia. Bone Marrow Transplant.

[5] Larone DH (2011). Medically Important Fungi: A Guide to Identification.

[6] Murray PR, Rosenthal KS, Pfaller MA (2013). Medical Microbiology.

[7] Hazen KC, Howell SA, Garcia LS (2007). Mycology and antifungal susceptibility testing. Clinical Microbiology Procedures Handbook.

[8] Perfect JR, Cox GM, Lee JY, Kauffman CA, Repentigny L, Chapman SW, Morrison VA, Pappas P, Hiemenz JW (2001). The impact of culture isolation of Aspergillus species: a hospital-based survey of aspergillosis. Clin Infect Dis.

[9] Horvath JA, Dummer S (1996). The use of respiratory-tract cultures in the diagnosis of invasive pulmonary aspergillosis. Am J Med.

[10] Clancy CJ, Nguyen MH (2013). Finding the “missing 50%” of invasive candidiasis: how nonculture diagnostics will improve understanding of disease spectrum and transform patient care. Clin Infect Dis.

[11] Sims CR, Ostrosky-Zeichner L, Rex JH (2005). Invasive candidiasis in immunocompromised hospitalized patients. Arch Med Res.

[12] CLSI (2012). Principles and Procedures for Detection of Fungi in Clinical Specimens, Direct Examination and Culture; Approved Guideline, CLSI Document M54-A.. Wayne, PA, USA, CLSI.

[13] Creger RJ, Weeman KE, Jacobs MR, Morrissey A, Parker P, Fox RM, Lazarus HM (1998). Lack of utility of the lysis-centrifugation blood culture method for detection of fungemia in immunocompromised cancer patients. J Clin Microbiol.

[14] Chiarini A, Palmeri A, Amato T, Immordino R, Distefano S, Giammanco A (2008). Detection of bacterial and yeast species with the Bactec 9120 automated system with routine use of aerobic, anaerobic, and fungal media. J Clin Microbiol.

[15] Kirby JE, Delaney M, Qian Q, Gold HS (2009). Optimal use of Myco/F lytic and standard BACTEC blood culture bottles for detection of yeast and mycobacteria. Arch Pathol Lab Med.

[16] Arendrup MC, Bruun B, Christensen JJ, Fuursted K, Johansen HK, Kjaeldgaard P, Knudsen JD, Kristensen L, Møller J, Nielsen L, Rosenvinge FS, Røder B, Schønheyder HC, Thomsen MK, Truberg K (2011). National surveillance of fungemia in Denmark (2004 to 2009). J Clin Microbiol.

[17] Baron EJ, Miller JM, Weinstein MP, Richter SS, Gilligan PH, Thomson RB, Bourbeau P, Carroll KC, Kehl SC, Dunne WM, Robinson-Dunn B, Schwartzman JD, Chapin KC, Snyder JW, Forbes BA, Patel R, Rosenblatt JE, Pritt BS (2013). A guide to utilization of the microbiology laboratory for diagnosis of infectious diseases: 2013 recommendations by the Infectious Diseases Society of America (IDSA) and the American Society for Microbiology (ASM). Clin Infect Dis.

[18] CLSI (2008). Reference Method for Broth Dilution Antifungal Susceptibility Testing of Yeasts; Approved Standard-Third Edition. CLSI Document M27-A3. Wayne, PA, USA, CLSI.

[19] CLSI (2008). Reference Method for Broth Dilution Antifungal Susceptibility Testing of Filamentous Fungi; Approved Standard-Second Edition. CLSI Document M38-A2. Wayne, PA, USA, CLSI,.

[20] Arendrup MC, Cuenca-Estrella M, Lass-Flörl C, Hope W, EUCAST-AFST (2012). EUCAST technical note on the EUCAST definitive document EDef 7.2: method for the determination of broth dilution minimum inhibitory concentrations of antifungal agents for yeasts EDef 7.2 (EUCAST-AFST).. Clin Microbiol Infect.

[21] No Authors (2008). Subcommittee on Antifungal Susceptibility Testing of the ESCMID European Committee for Antimicrobial Susceptibility Testing, Technical Note on the method for the determination of broth dilution minimum inhibitory concentrations of antifungal agents for conidia-forming moulds. Clin Microbiol Infect.

[22] Marchetti O, Lamoth F, Mikulska M, Viscoli C, Verweij P (2012). ECIL recommendations for the use of biological markers for the diagnosis of invasive fungal diseases in leukemic patients and hematopoietic SCT recipients. Bone Marrow Transplant.

[23] Marchetti O, Lamoth F, Mikulska M, Viscoli C, Verweij P (2012). ECIL recommendations for the use of biological markers for the diagnosis of invasive fungal diseases in leukemic patients and hematopoietic SCT recipients. Bone Marrow Transplant.

[24] Chowdhary A, Meis JF, Guarro J, Hoog GS, Kathuria S, Arendrup MC, Arikan-Akdagli S, Akova M, Boekhout T, Caira M, Guinea J, Chakrabarti A, Dannaoui E, Diepeningen A, Freiberger T, Groll AH, Hope WW, Johnson E, Lackner M, Lagrou K, Lanternier F, Lass-Flörl C, Lortholary O, Meletiadis J, Muñoz P, Pagano L, Petrikkos G, Richardson MD, Roilides E, Skiada A, Tortorano AM, Ullmann AJ, Verweij PE, Cornely OA, Cuenca-Estrella M (2014). ESCMID and ECMM joint clinical guidelines for the diagnosis and management of systemic phaeohyphomycosis: diseases caused by black fungi. Clin Microbiol Infect.

[25] Tortorano AM, Richardson M, Roilides E, Diepeningen A, Caira M, Munoz P, Johnson E, Meletiadis J, Pana ZD, Lackner M, Verweij P, Freiberger T, Cornely OA, Arikan-Akdagli S, Dannaoui E, Groll AH, Lagrou K, Chakrabarti A, Lanternier F, Pagano L, Skiada A, Akova M, Arendrup MC, Boekhout T, Chowdhary A, Cuenca-Estrella M, Guinea J, Guarro J, Hoog S, Hope W, Kathuria S, Lortholary O, Meis JF, Ullmann AJ, Petrikkos G, Lass-Flörl C (2014). ESCMID and ECMM joint guidelines on diagnosis and management of hyalohyphomycosis: Fusarium spp, Scedosporium spp. and others. Clin Microbiol Infect.

[26] Cornely OA, Arikan-Akdagli S, Dannaoui E, Groll AH, Lagrou K, Chakrabarti A, Lanternier F, Pagano L, Skiada A, Akova M, Arendrup MC, Boekhout T, Chowdhary A, Cuenca-Estrella M, Freiberger T, Guinea J, Guarro J, Hoog S, Hope W, Johnson E, Kathuria S, Lackner M, Lass-Flörl C, Lortholary O, Meis JF, Meletiadis J, Muñoz P, Richardson M, Roilides E, Tortorano AM, Ullmann AJ, Diepeningen A, Verweij P, Petrikkos G (2014). ESCMID and ECMM joint clinical guidelines for the diagnosis and management of mucormycosis 2013. Clin Microbiol Infect.

[27] Arendrup MC, Boekhout T, Akova M, Meis JF, Cornely OA (2014). ESCMID and ECMM joint clinical guidelines for the diagnosis and management of rare invasive yeast infections. Clin Microbiol Infect.

[28] Latge JP, Kobayashi H, Debeaupuis JP, Diaquin M, Sarfati J, Wieruszeski JM, Parra E, Bouchara JP, Fournet B (1994). Chemical and immunological characterization of the extracellular galactomannan of Aspergillus fumigatus. Infect Immun.

[29] Swanink CM, Meis JF, Rijs AJ, Donnelly JP, Verweij PE (1997). Specificity of a sandwich enzyme-linked immunosorbent assay for detecting Aspergillus galactomannan. J Clin Microbiol.

[30] Maertens J, Verhaegen J, Lagrou K, Van Eldere J, Boogaerts M (2001). Screening for circulating galactomannan as a noninvasive diagnostic tool for invasive aspergillosis in prolonged neutropenic patients and stem cell transplantation recipients: a prospective validation. Blood.

[31] Kawazu M, Kanda Y, Nannya Y, Aoki K, Kurokawa M, Chiba S, Motokura T, Hirai H, Ogawa S (2004). Prospective comparison of the diagnostic potential of real-time PCR, double-sandwich enzyme-linked immunosorbent assay for galactomannan, and a (1→3)-beta-D-glucan test in weekly screening for invasive aspergillosis in patients with hematological disorders. J Clin Microbiol.

[32] Musher B, Fredricks D, Leisenring W, Balajee SA, Smith C, Marr KA (2004). Aspergillus galactomannan enzyme immunoassay and quantitative PCR for diagnosis of invasive aspergillosis with bronchoalveolar lavage fluid. J Clin Microbiol.

[33] Klont RR, Mennink-Kersten MA, Verweij PE (2004). Utility of Aspergillus antigen detection in specimens other than serum specimens. Clin Infect Dis.

[34] Koo S, Bryar JM, Baden LR, Marty FM (2010). Prognostic features of galactomannan antigenemia in galactomannan-positive invasive aspergillosis. J Clin Microbiol.

[35] De Pauw B, Walsh TJ, Donnelly JP, Stevens DA, Edwards JE, Calandra T, Pappas PG, Maertens J, Lortholary O, Kauffman CA, Denning DW, Patterson TF, Maschmeyer G, Bille J, Dismukes WE, Herbrecht R, Hope WW, Kibbler CC, Kullberg BJ, Marr KA, Muñoz P, Odds FC, Perfect JR, Restrepo A, Ruhnke M, Segal BH, Sobel JD, Sorrell TC, Viscoli C, Wingard JR, Zaoutis T (2008). Revised definitions of invasive fungal disease from the European Organization for Research and Treatment of Cancer/Invasive Fungal Infections Cooperative Group and the National Institute of Allergy and Infectious Diseases Mycoses Study Group (EORTC/MSG) Consensus Group. Clin Infect Dis.

[36] Marr KA, Balajee SA, McLaughlin L, Tabouret M, Bentsen C, Walsh TJ (2004). Detection of galactomannan antigenemia by enzyme immunoassay for the diagnosis of invasive aspergillosis: variables that affect performance. J Infect Dis.

[37] Marr KA, Laverdiere M, Gugel A, Leisenring W (2005). Antifungal therapy decreases sensitivity of the Aspergillus galactomannan enzyme immunoassay. Clin Infect Dis.

[38] Pfeiffer CD, Fine JP, Safdar N (2006). Diagnosis of invasive aspergillosis using a galactomannan assay: a meta-analysis. Clin Infect Dis.

[39] Hachem RY, Kontoyiannis DP, Chemaly RF, Jiang Y, Reitzel R, Raad I (2009). Utility of galactomannan enzyme immunoassay and (1,3) beta-D-glucan in diagnosis of invasive fungal infections: low sensitivity for Aspergillus fumigatus infection in hematologic malignancy patients. J Clin Microbiol.

[40] Kebabci N, Diepeningen AD, Ener B, Ersal T, Meijer M, Al-Hatmi AM, Ozkocaman V, Ursavaş A, Cetinoğlu ED, Akalın H (2014). Fatal breakthrough infection with Fusarium andiyazi: new multi-resistant aetiological agent cross-reacting with Aspergillus galactomannan enzyme immunoassay. Mycoses.

[41] Metan G, Agkus C, Buldu H, Koç AN (2010). The interaction between piperacillin/tazobactam and assays for Aspergillus galactomannan and 1,3-beta-D-glucan in patients without risk factors for invasive fungal infections. Infection.

[42] Mikulska M, Furfaro E, Del Bono V, Raiola AM, Ratto S, Bacigalupo A, Viscoli C (2012). Piperacillin/tazobactam (Tazocin™) seems to be no longer responsible for false-positive results of the galactomannan assay. J Antimicrob Chemother.

[43] King ST, Stover KR (2014). Considering confounders of the galactomannan index: the role of piperacillin-tazobactam. Clin Infect Dis.

[44] Orlopp K, von Lilienfeld-Toal M, Marklein G, Reiffert SM, Welter A, Hahn-Ast C, Purr I, Gorschlüter M, Molitor E, Glasmacher A (2008). False positivity of the Aspergillus galactomannan Platelia ELISA because of piperacillin/tazobactam treatment: does it represent a clinical problem?. J Antimicrob Chemother.

[45] Heng SC, Morrissey O, Chen SC, Thursky K, Manser RL, Nation RL, Kong DC, Slavin M (2013). Utility of bronchoalveolar lavage fluid galactomannan alone or in combination with PCR for the diagnosis of invasive aspergillosis in adult hematology patients: a systematic review and meta-analysis. Crit Rev Microbiol.

[46] Zou M, Tang L, Zhao S, Zhao Z, Chen L, Chen P, Huang Z, Li J, Chen L, Fan X (2012). Systematic review and meta-analysis of detecting galactomannan in bronchoalveolar lavage fluid for diagnosing invasive aspergillosis. PLoS One.

[47] Guo YL, Chen YQ, Wang K, Qin SM, Wu C, Kong JL (2010). Accuracy of BAL galactomannan in diagnosing invasive aspergillosis: a bivariate metaanalysis and systematic review. Chest.

[48] Maertens J, Maertens V, Theunissen K, Meersseman W, Meersseman P, Meers S, Verbeken E, Verhoef G, Van Eldere J, Lagrou K (2009). Bronchoalveolar lavage fluid galactomannan for the diagnosis of invasive pulmonary aspergillosis in patients with hematologic diseases. Clin Infect Dis.

[49] D’Haese J, Theunissen K, Vermeulen E, Schoemans H, De Vlieger G, Lammertijn L, Meersseman P, Meersseman W, Lagrou K, Maertens J (2012). Detection of galactomannan in bronchoalveolar lavage fluid samples of patients at risk for invasive pulmonary aspergillosis: analytical and clinical validity. J Clin Microbiol.

[50] Ağca H, Ener B, Yılmaz E, Ursavaş A, Kazak E, Özkocaman V, Çetinoğlu ED, Dilektaşlı AG, Akalın H, Özkalemkaş F, Ali R (2014). Comparative evaluation of galactomannan optical density indices and culture results in bronchoscopic specimens obtained from neutropenic and non-neutropenic patients. Mycoses.

[51] Odabasi Z, Mattiuzzi G, Estey E, Kantarjian H, Saeki F, Ridge RJ, Ketchum PA, Finkelman MA, Rex JH, Ostrosky-Zeichner L (2004). Beta-D-glucan as a diagnostic adjunct for invasive fungal infections: validation, cutoff development, and performance in patients with acute myelogenous leukemia and myelodysplastic syndrome. Clin Infect Dis.

[52] Obayashi T, Kawai T, Yoshida M, Mori T, Goto H, Yasuoka A, Shimada K, Iwasaki H, Teshima H, Kohno S, Horiuchi A, Ito A, Yamaguchi H (1995). Plasma (1→3)-β-D-glucan measurement in diagnosis of invasive deep mycosis and fungal febrile episodes. Lancet.

[53] Ostrosky-Zeichner L, Alexander BD, Kett DH, Vazquez J, Pappas PG, Saeki F, Ketchum PA, Wingard J, Schiff R, Tamura H, Finkelman MA, Rex JH (2005). Multicenter clinical evaluation of the (1→3) β-D-glucan assay as an aid to diagnosis of fungal infections in humans. Clin Infect Dis.

[54] Odabasi Z, Paetznick VL, Rodriguez JR, Chen E, McGinnis MR, Ostrosky-Zeichner L (2006). Differences in beta-glucan levels in culture supernatants of a variety of fungi. Med Mycol.

[55] Onishi A, Sugiyama D, Kogata Y, Saegusa J, Sugimoto T, Kawano S, Morinobu A, Nishimura K, Kumagai S (2012). Diagnostic accuracy of serum 1,3-β-D-glucan for Utility of bronchoalveolar lavage fluid galactomannan alone or in combination with PCR for the diagnosis of invasive aspergillosis in adult hematology patients: a systematic review and meta-analysis pneumonia, invasive candidiasis, and invasive aspergillosis: systematic review and meta-analysis. J Clin Microbiol.

[56] Theel ES, Jespersen DJ, Iqbal S, Bestrom JE, Rollins LO, Misner LJ, Markley BJ, Mandrekar J, Baddour LM, Limper AH, Wengenack NL, Binnicker MJ (2013). Detection of (1, 3)-β-D-glucan in bronchoalveolar lavage and serum samples collected from immunocompromised hosts. Mycopathologia.

[57] Pazos C, Pontón J, Del Palacio A (2005). Contribution of (1→3)-β-D-glucan chromogenic assay to diagnosis and therapeutic monitoring of invasive aspergillosis in neutropenic adult patients: a comparison with serial screening for circulating galactomannan. J Clin Microbiol.

[58] Karageorgopoulos DE, Vouloumanou EK, Ntziora F, Michalopoulos A, Rafailidis PI, Falagas ME (2011). β-D-glucan assay for the diagnosis of invasive fungal infections: a meta-analysis. Clin Infect Dis.

[59] Lamoth F, Cruciani M, Mengoli C, Castagnola E, Lortholary O, Richardson M (2012). β-Glucan antigenemia assay for the diagnosis of invasive fungal infections in patients with hematological malignancies: a systematic review and meta-analysis of cohort studies from the Third European Conference on Infections in Leukemia (ECIL-3). Clin Infect Dis.

[60] Hoenigl M, Prattes J, Spiess B, Wagner J, Prueller F, Raggam RB, Posch V, Duettmann W, Hoenigl K, Wölfler A, Koidl C, Buzina W, Reinwald M, Thornton CR, Krause R, Buchheidt D (2014). Performance of galactomannan, beta-D-glucan, Aspergillus lateral-flow device, conventional culture and PCR tests for diagnosis of invasive pulmonary aspergillosis in bronchoalveolar lavage fluid. J Clin Microbiol.

[61] Atalay A, Metan G, Koc AN, Kaynar LG, Buyukoglan H, Bozkurt I, Yildirim A, Elmali F, Eser B (2012). Detection of (1,3)-beta-D-glucan In: Bronchoalveolar Lavage Fluid for the Diagnosis of Invasive Aspergillosis. 52nd Interscience Congress of Antimicrobial Chemotherapy.

[62] Babady NE, Bestrom JE, Jespersen DJ, Jones MF, Beito EM, Binnicker MJ, Wengenack NL (2009). Evaluation of three commercial latex agglutination kits and a commercial enzyme immunoassay for the detection of cryptococcal antigen. Med Mycol.

[63] Chayakulkeeree M, Perfect JR (2006). Cryptococcosis. Infect Dis Clin North Am.

[64] Husain S, Wagener MM, Singh N (2001). Cryptococcus neoformans infection in organ transplant recipients: variables influencing clinical characteristics and outcome. Emerg Infect Dis.

[65] Dromer F, Mathoulin-Pélissier S, Launay O (2007). Determinants of disease presentation and outcome during cryptococcosis: the CryptoA/D study. PLoS Med.

[66] Pappas PG, Perfect JR, Cloud GA, Larsen RA, Pankey GA, Lancaster DJ, Henderson H, Kauffman CA, Haas DW, Saccente M, Hamill RJ, Holloway MS, Warren RM, Dismukes WE (2001). Cryptococcosis in human immunodeficiency virus-negative patients in the era of effective azole therapy. Clin Infect Dis.

[67] Jongwutiwes U, Sungkanuparph S, Kiertiburanakul S (2008). Comparison of clinical features and survival between cryptococcosis in human immunodeficiency virus (HIV)-positive and HIV-negative patients. Jpn J Infect Dis.

[68] Tanner DC, Weinstein MP, Fedorciw B, Joho KL, Thorpe JJ, Reller L (1994). Comparison of commercial kits for detection of cryptococcal antigen. J Clin Microbiol.

[69] Kontoyiannis DP (2003). What is the significance of an isolated positive cryptococcal antigen in the cerebrospinal fluid of cancer patients?. Mycoses.

[70] Mikulska M, Calandra T, Sanguinetti M, Poulain D (2010). The use of mannan antigen and anti-mannan antibodies in the diagnosis of invasive candidiasis: recommendations from the Third European Conference on Infections in Leukemia. Crit Care.

[71] Yera H, Sendid B, Francois N, Camus D, Poulain D (2001). Contribution of serological tests and blood culture to the early diagnosis of systemic candidiasis. Eur J Clin Microbiol Infect Dis.

[72] Prella M, Bille J, Pugnale M, Duvoisin B, Cavassini M, Calandra T, Marchetti O (2005). Early diagnosis of invasive candidiasis with mannan antigenemia and antimannan antibodies. Diagn Microbiol Infect Dis.

[73] Ellis M, Al-Ramadi B, Bernsen R, Kristensen J, Alizadeh H, Hedstrom U (2009). Prospective evaluation of mannan and anti-mannan antibodies for diagnosis of invasive Candida infections in patients with neutropenic fever. J Med Microbiol.

[74] Held J, Kohlberger I, Rappold E, Busse Grawitz A, Häcker G (2013). Comparison of (1→3)-β-D-glucan, mannan/anti-mannan antibodies, and Cand-Tec Candida antigen as serum biomarkers for candidemia. J Clin Microbiol.

[75] Marom EM, Kontoyiannis DP (2011). Imaging studies for diagnosing invasive fungal pneumonia in immunocompromised patients. Curr Opin Infect Dis.

[76] Franquet T, Giménez A, Hidalgo A (2004). Imaging of opportunistic fungal infections in immunocompromised patient. Eur J Radiol.

[77] Chong S, Lee KS, Yi CA, Chung MJ, Kim TS, Han J (2006). Pulmonary fungal infection: imaging findings in immunocompetent and immunocompromised patients. Eur J Radiol.

[78] Hansell DM, Bankier AA, MacMahon H, McLoud TC, Müller NL, Remy J (2008). Fleischner Society: glossary of terms for thoracic imaging. Radiology.

[79] Brodoefel H, Vogel M, Hebart H, Einsele H, Vonthein R, Claussen C, Horger M (2006). Long-term CT follow-up in 40 non-HIV immunocompromised patients with invasive pulmonary aspergillosis: kinetics of CT morphology and correlation with clinical findings and outcome. Am J Roentgenol.

[80] Caillot D, Couaillier JF, Bernard A, Casasnovas O, Denning DW, Mannone L, Lopez J, Couillault G, Piard F, Vagner O, Guy H (2001). Increasing volume and changing characteristics of invasive pulmonary aspergillosis on sequential thoracic computed tomography scans in patients with neutropenia. J Clin Oncol.

[81] Wahba H, Truong MT, Lei X, Kontoyiannis DP, Marom EM (2008). Reversed halo sign in invasive pulmonary fungal infections. Clin Infect Dis.

[82] Ascioglu S, Rex JH, De Pauw B, Bennett JE, Bille J, Crokaert F, Denning DW, Donnelly JP, Edwards JE, Erjavec Z, Fiere D, Lortholary O, Maertens J, Meis JF, Patterson TF, Ritter J, Selleslag D, Shah PM, Stevens DA (2002). Defining opportunistic invasive fungal infections in immunocompromised patients with cancer and hematopoietic stem cell transplants: an international consensus. Clin Infect Dis.

